# Quantitative Single-letter Sequencing: a method for simultaneously monitoring numerous known allelic variants in single DNA samples

**DOI:** 10.1186/1471-2164-9-85

**Published:** 2008-02-21

**Authors:** Baptiste Monsion, Hervé Duborjal, Stéphane Blanc

**Affiliations:** 1 Biologie et Génétique des Interactions Plante-Parasite (BGPI), INRA-CIRAD-SupagroM, TA A-54/K, Campus International de Baillarguet, 34398 Montpellier Cedex 5, France; 2 COGENICS GENOME Express SA, 38944 Meylan, France

## Abstract

**Background:**

Pathogens such as fungi, bacteria and especially viruses, are highly variable even within an individual host, intensifying the difficulty of distinguishing and accurately quantifying numerous allelic variants co-existing in a single nucleic acid sample. The majority of currently available techniques are based on real-time PCR or primer extension and often require multiplexing adjustments that impose a practical limitation of the number of alleles that can be monitored simultaneously at a single locus.

**Results:**

Here, we describe a novel method that allows the simultaneous quantification of numerous allelic variants in a single reaction tube and without multiplexing. Quantitative Single-letter Sequencing (QSS) begins with a single PCR amplification step using a pair of primers flanking the polymorphic region of interest. Next, PCR products are submitted to single-letter sequencing with a fluorescently-labelled primer located upstream of the polymorphic region. The resulting monochromatic electropherogram shows numerous specific diagnostic peaks, attributable to specific variants, signifying their presence/absence in the DNA sample. Moreover, peak fluorescence can be quantified and used to estimate the frequency of the corresponding variant in the DNA population.

Using engineered allelic markers in the genome of *Cauliflower mosaic virus*, we reliably monitored six different viral genotypes in DNA extracted from infected plants. Evaluation of the intrinsic variance of this method, as applied to both artificial plasmid DNA mixes and viral genome populations, demonstrates that QSS is a robust and reliable method of detection and quantification for variants with a relative frequency of between 0.05 and 1.

**Conclusion:**

This simple method is easily transferable to many other biological systems and questions, including those involving high throughput analysis, and can be performed in any laboratory since it does not require specialized equipment.

## Background

The need to analyze genetic variation within populations of various organisms has engendered a wide variety of techniques designed to identify genetic differences between related genomes [1,2]. The vast majority of methods currently available for detecting single or polynucleotide polymorphisms, as well as insertions and deletions of varying size, were originally developed for genotyping individual organisms within populations. By sampling and genotyping numerous separate individuals, the relative frequency of a given variant within a population can be estimated. However, the number of samples analyzed rapidly becomes a limiting factor, in terms of both time and cost considerations. As a consequence, a new generation of techniques is being developed that will allow more than one specific allele to be distinguished and quantified simultaneously [3-6] in a single sample containing nucleic acids pooled from several individuals [7,8]. The most successful techniques described so far have various advantages and drawbacks. On the one hand, techniques based on microarrays [9,10] or on mass spectrometry [11-14], although very efficient at sorting numerous variants within a single sample, require access to specialized equipment and are thus difficult and expensive to develop and implement. On the other hand, more amenable techniques based on real-time PCR or primer extension [15,16] often require multiplexing adjustments.

For pathogenic microorganisms, such as fungi, bacteria and especially viruses, populations infecting an individual host are often highly variable, intensifying the difficulties involved in distinguishing and accurately quantifying numerous alleles or variants co-existing in a single nucleic acid sample. This is best illustrated by considering the properties of viral populations, which exist as a swarm of related mutants commonly designated as a quasispecies [17]. One remarkable property of viral quasispecies is that the relative frequency and distribution of specific variants can vary at different phases of the virus life cycle [18]. In this situation, the ability to simultaneously monitor those specific variants can become critical in understanding the biology and evolution of the virus [19]. Similarly, monitoring changes in allele frequency is essential to the understanding of the evolution of viruses or other pathogens, as these changes reflect forces such as selection during adaptation [20] and genetic drift in phases of the life cycle where the effective population size is low [21,22].

We faced exactly this challenge when evaluating the effective population size of *Cauliflower mosaic virus *(CaMV) during systemic invasion of plant host tissues (to be described elsewhere). For this purpose, and because of concerns related to the available genotyping techniques mentioned above, we developed a novel analysis method based on classical detection of genetic variants by dideoxy fingerprinting [23,24]. This method, named Quantitative Single-letter Sequencing (QSS), allowed the simultaneous and accurate monitoring of six engineered allelic CaMV variants over time, determining in a single nucleic acid sample and in a single reaction process both the presence/absence and the relative frequency of each allele in the viral genome population.

Here, we describe the QSS method and its application to samples consisting of mixtures of purified plasmids or viral genomes extracted from a multiply infected host plant. We test the accuracy, reproducibility, robustness and limits of this method, and discuss its potential application to other biological systems in any standard laboratory setting. Since QSS applies to known sequences only, it will be useful for monitoring experimental populations, as well as natural populations where the different variants at the targeted locus have been previously identified.

## Results

### Single-letter sequencing distinguishes several markers in mixed plasmid solutions

**Figure 1:**
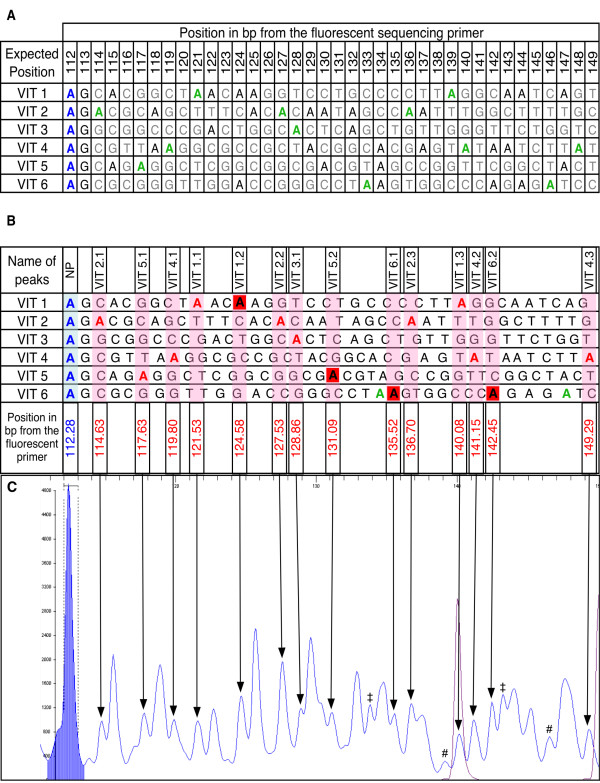
**Sequence signatures identify different markers in a mixed DNA solution**. (A) Sequences of the six genetic markers pCaVIT-1 to -6. The blue A on the left is the last common adenine shared between all pCa-VIT1-6 plasmids upstream of the polymorphic region. Adenine residues marked in green are theoretically expected to yield discriminating peaks; ther A residues are indicated in black. The expected position (in bp) of each base from the fluorescent sequencing primer is indicated at the top. (B) Experimentally observed positions of discriminating A-peaks (named VIT1.1 to VIT6.2 at the top) on sequence traces (not shown) from individually sequenced markers. Sequences of all six markers are slightly distorted in order to match the actual position of A peaks observed on individual electropherograms. The red A residues were each confirmed to yield a discriminating peak. Though not theoretically predicted to do so, the red-boxed As proved to yield discriminating peaks experimentally. The remaining green As failed to produce discriminating-peaks. Sequencing reactions of pCa-VIT1-6 were repeated three times, and the observed position of all peaks was highly reproducible: +/- 0.2 bp among repeats. At the bottom, the number in blue is the average position of the last A common to all sequences upstream of the differential markers. The numbers in red are the average positions of the observed discriminating peaks. (C) Single-letter Sequencing electropherogram from a mixed DNA solution containing all 6 markers. A mix of all 6 pCaVIT1-6 plasmids in equal amounts was used as a template for PCR and subsequent single-letter sequencing. All discriminating-peaks defined in B are indicated by arrows on the electropherogram. The shaded blue peak corresponds to the last common A. The scale at the top indicates the nucleotide position relative to that of the sequencing primer. The scale on the left is used for scoring the height of the peaks in arbitrary units provided by the STRand program. The two purple peaks correspond to molecular weight markers. # These peaks are artefacts. ‡ Though appearing as discriminating, these peaks actually overlap small artefactual peaks observed on at least one electropherogram from individually sequenced markers (not shown). They are thus not used further.

Genetic markers were engineered into the CaMV genomes at exactly the same position. Thus, examining single letter (A) sequence traces from a mixed population should, in theory, reveal some common peaks at positions where more than one marker harbours a A, and discriminating peaks at positions where only one marker does so. Figure 1A shows an alignment of the marker sequences, highlighting the theoretically discriminating A-positions in green. Between one and three discriminating A-positions are expected for each of the 6 markers used in this study.

The actual observed positions of A-peaks from the amplified and single-letter-sequenced pCaVIT1-6 plasmids showed slight differences from their theoretical positions relative to a co-electrophoresed labeled DNA ladder (Figure 1B). Sequencing of each individual CaMV VIT clone was repeated three times, and the observed position of the A-peaks proved to be highly reproducible, with the position of each peak always occurring in the same place to within ± 0.2 bp (not shown). Therefore, we defined an experimental list of discriminating A-positions, using the actual observed peak positions rather than their original theoretically predicted positions, as explained and highlighted in red in Figure 1B.

This experimental list of discriminating A-positions was validated when all six markers co-existed in the DNA sample and were analyzed together. A mix of equal amounts of all pCaVIT1-6 plasmids was used as a template for PCR and subsequent single-letter sequencing. All experimentally-confirmed discriminating A-peaks were easily distinguished at their specific position on the electropherogram (Figure 1C), demonstrating that pooling plasmids in a single analysis did not lead to interference between the different markers. Hence, for each pCa-VIT plasmid, from one to three discriminating peaks can be reliably detected on the electropherogram and indicate the presence/absence of the corresponding marker, determining the qualitative composition of the DNA sample.

In all subsequent experiments, only those peaks that perfectly matched their expected position (± 0.2 bp of the position shown in Figure 1B) were considered reliable and included in the analysis.

### Standard curves for quantification from single-letter sequence traces

We used the last peak common to all sequences before the polymorphic region (shaded blue in Figure 1C) – called the normalizing-peak – to normalize the recorded data. The height of the normalizing-peak is contributed to by all the DNA molecules in the mixed solution, and is thus generated by 100% of the sequences in the analyzed sample. In contrast, the discriminating peaks are generated only by those sequences containing an A at the corresponding position. Each arrested DNA molecule produced by the sequencing process contains one single fluorochrome linked to the sequencing primer; hence the height of a discriminating-peak is proportional to the relative frequency of the corresponding marker in the DNA solution.

In order to prepare standard curves for the quantification of peak heights, we first prepared a series of mixed plasmid solutions containing pCa-VIT1 at a relative frequency of 5, 10, 15, 25, 50, 75 and 100%, maintaining equal amounts of the other five pCa-VIT plasmids in all but the 100% pCa-VIT solution. Similar mixed solutions were prepared for pCa-VIT2, pCa-VIT3, pCa-VIT4, pCa-VIT5 and pCa-VIT6 and processed for PCR amplification and single-letter sequencing as described in Methods.

**Figure 2:**
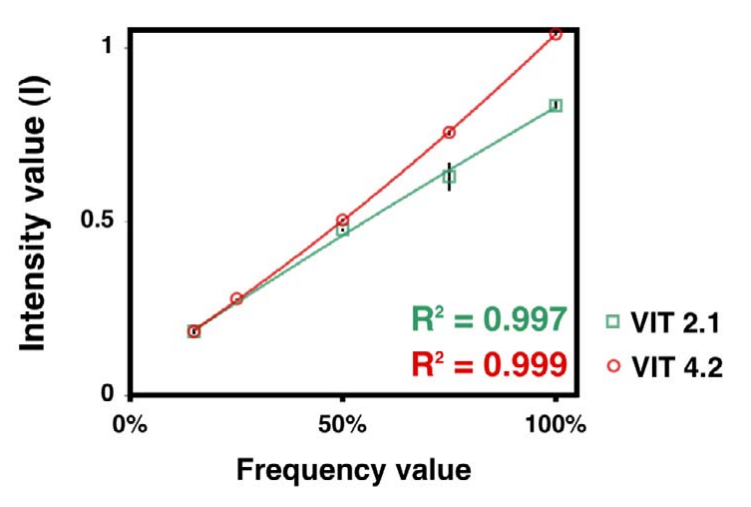
**Standard curves for converting the intensity of discriminating peaks into frequency of corresponding markers**. Standard curves were established for all 14 discriminating-peaks shown in Figure 1. Only those for VIT2.1 and VIT 4.2 are shown here, as they illustrate the worst and the best fit, respectively, between the recorded data and the polynomial regression function calculated using Excel (correlation coefficients R^2^, are shown). Vertical bars represent standard deviation among repeats.

To extract information from the electropherograms on the relative frequency of each marker present in a mixed solution, we assigned an intensity value (I) to each discriminating-peak, corresponding to its height relative to that of the normalizing-peak. For each given discriminating-peak, a standard curve was constructed by plotting the known frequencies of the corresponding marker against the observed I values (Figure 2). One standard curve for each discriminating-peak was established from 3 replicates at 100%, 2 replicates at 75, 50 and 25%, and 10 replicates at 15% of the corresponding marker in the mixed plasmid solution. In these replicate runs, the I values were highly reproducible and showed very limited variation (Figure 2). However, I values obtained at 10% and 5% were more variable and were not used in establishing the standard curves (mean standard deviations were 3 and 3.25%, respectively).

The polynomial regression function corresponding to each standard curve was calculated using Excel (Microsoft). The R^2 ^values obtained for all standard curves were remarkably high, and two examples (corresponding to curves with the highest and the lowest R^2 ^values) are shown in Figure 2.

### Accuracy of quantification of genetic markers within mixed DNA populations

The standard curves shown in Figure 2 were used to directly transform the observed I values into the frequency of the corresponding marker in test DNA solutions. Parallel estimates were possible when more than one discriminating-peak was available for a given marker. In such cases, the frequency was taken as the mean of the parallel estimates.

**Figure 3:**
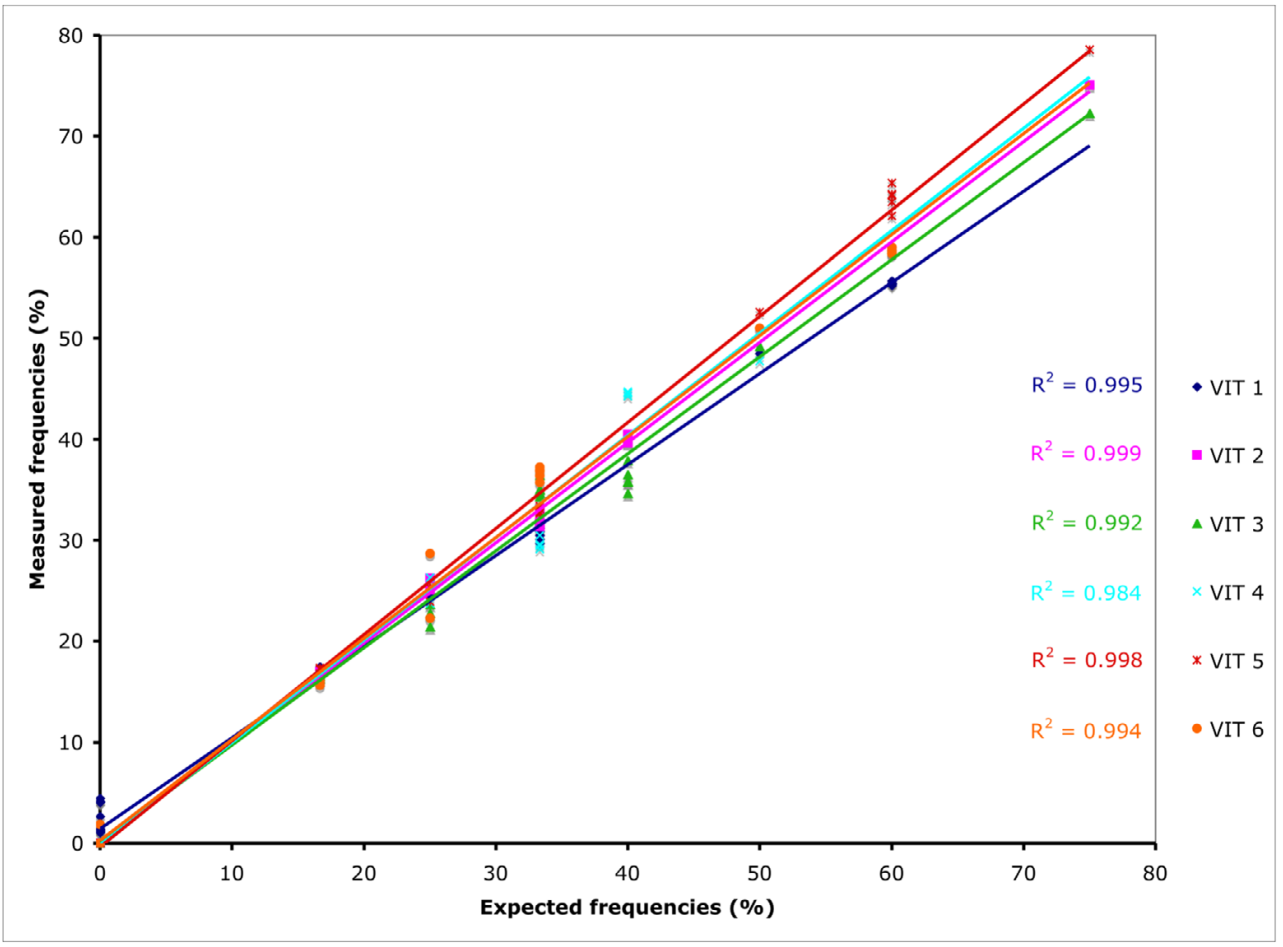
**Accuracy of QSS measurements for the 6 genetic markers**. Observed values are plotted against expected values for all measurements summarized in Table 3, plus four independent repeats of the analysis of mixes N°9 to 14. For each VIT marker, a linear regression function was deduced (colored lines). The near perfect scores for the R^2 ^correlation coefficient in each case illustrate the high degree of accuracy of QSS.

The accuracy of the quantification was assessed by comparing the measured frequencies to their expected values on plasmid mixes prepared from the reference stocks. Several mixes were tested (compositions listed in Table 1). Whether the mixes contained only 2 or all 6 markers, QSS yielded estimates remarkably close to the expected values for all markers tested (Table 2). Surprisingly, when the estimates for each marker present in a given solution were summed up, the total often deviated from 100%, ranging from about 90 to 120% (Table 2). This phenomenon was attributed to fluctuations in the baseline of sequence traces and was corrected by proportionally adjusting all marker frequencies to give a total of 100% (Table 3). This final correction yielded estimates even closer to the true marker frequencies (discussed further below). The overall accuracy of the method is illustrated in Figure 3, which shows the linear regression function correlating expected values to the estimates. The R^2 ^correlation coefficient was excellent for all VIT markers, being between 0.984 and 0.999.

**Table 1:** Composition of plasmid DNA mixes

	**Percentage of each VIT**					
	
**Mix n°**	***VIT1***	***VIT2***	***VIT3***	***VIT4***	***VIT5***	***VIT6***
1		75				25
2			75	25		
3			25		75	
4	50		25			25
5	25	25	50			
6	25			50	25	
7			25	25	50	
8		25			25	50
9	16.67	16.67	16.67	16.67	16.67	16.67
10	33.33				33.33	33.33
11		33.33	33.33	33.33		
12			40		60	
13		40				60
14	60			40		

Different mixes containing some or all pCa-VIT plasmids in the proportions indicated.

**Table 2:** Accuracy of QSS on mixtures of plasmid DNA before corrections

	**Percentage of each VIT**						**Total (%)**
		
**Mix n°**	***VIT1***	***VIT2***	***VIT3***	***VIT4***	***VIT5***	***VIT6***	
1		74.8				22.2	97
2			78.02	28.5			106.52
3			19.4		71.1		90.5
4	50		23.5			29.6	103.1
5	27.3	29.1	54.6				111
6	27.8			52.9	28.1		108.8
7			23.3	23.4	51.7		98.4
8		22.7			21.6	46.2	90.5
9	17	16.9	16.6	16.8	16.7	16.5	100.5
10	30.3				32.1	34.6	97
11		36.4	40.1	33.5			110
12			36.5		59.8		96.3
13		39.2				58.3	97.5
14	66.8			53.1			119.9

Marker frequency quantification, obtained from the standard curves shown in Figure 2. The sum of frequencies in each mix is shown on the right.

**Table 3:** Accuracy of QSS on mixtures of plasmid DNA after final corrections

	**Percentage of each VIT**						**Total (%)**
		
**Mix n°**	***VIT1***	***VIT2***	***VIT3***	***VIT4***	***VIT5***	***VIT6***	
1		77.1				22.9	100
2			73.3	26.7			100
3			21.4		78.6		100
4	48.5		22.8			28.7	100
5	24.6	26.2	49.2				100
6	25.6			48.6	25.8		100
7			23.7	23.7	52.6		100
8		25.1			23.9	51	100
9	16.9	16.8	16.6	16.7	16.6	16.4	100
10	31.2				33.1	35.7	100
11		33.1	36.4	30.5			100
12			37.9		62.1		100
13		40.2				59.8	100
14	55.7			44.3			100

Frequency values after proportional correction to give a total of 100% in each mix.

The name Quantitative Single-letter Sequencing (QSS) is used to describe the overall process of identifying discriminating peaks, recording their normalized height, and converting them into frequencies of genetic markers in a mixed DNA population.

### Reproducibility of QSS in repeated analyses of plasmids and virus mixes

We performed five independent PCR-amplifications of plasmid mix N°9 (Table 1), containing equal amounts of all six markers, and further performed QSS analyses. In all five independent repeats, the estimated frequency of each marker (Table 4), ranging from 15.62 to 17.45%, was very close to the expected value of 16.6%. The standard deviation among repeats was very small (< 0.5%) for all markers considered (median of the 6 SD = 0.281%), confirming the high reproducibility of the process.

**Table 4:** Reproducibility of QSS on mixtures of plasmid DNA

	**Percentage of each VIT**						**Total (%)**
		
**Replicates of Mix N°9**	***VIT1***	***VIT2***	***VIT3***	***VIT4***	***VIT5***	***VIT6***	
1	16.92	16.78	16.55	16.72	16.59	16.43	100
2	17.45	16.99	17.14	16.12	16.19	16.12	100
3	17.17	16.58	16.81	16.74	16.79	15.92	100
4	17.06	16.47	16.32	16.68	17.33	16.14	100
5	17.19	16.36	17.03	16.60	17.21	15.62	100
Mean	17,16	16,63	16,77	16,57	16,82	16,05	100
SD	0.195	0.251	0.336	0.261	0.465	0.301	

Reproducibility of QSS as evaluated by the standard deviation (SD) on 5 replications of the analysis of Mix n°9. Proportional correction of the frequency of all markers was applied to give a total of 100% in each mix.

A similar reproducibility evaluation was also performed on a more realistic biological sample. A mixed population of viral DNA genomes was extracted from a single infected plant, originally inoculated with the CaMV Mix6VIT virus suspension as described in Methods. The extracted viral DNA was used in 19 independent PCR amplifications followed by QSS analysis (Table 5). Several conclusions can be drawn from the results of this experiment. i) QSS can be applied to "natural" samples, e.g. viral DNA solutions extracted from infected plants. ii) QSS is highly reproducible if genetic variants are sufficiently frequent in the population (above 5%), as the standard deviation among numerous repeats was very small. iii) The fact that the 6 markers were present in widely varying amounts allowed evaluation of the minimum frequency that can be reliably measured by QSS. When considering the standard deviation calculated for each marker among the 19 repetitions, it is clear that the method can efficiently detect and reliably quantify genetic variants present in the population at a frequency above 5% (Markers VIT1-4). This 5% frequency appears as the threshold for QSS reliability, both because corresponding variance between repeated measures proved too high when establishing the standard curves (see above), and because rarer markers (VIT5 and VIT6) yielded discriminating-peaks close to the sequencing baseline and could not be exploited unequivocally. iv) Finally, any variation observed for a given marker among the repeated experiments was only slightly affected by the quality of sequence traces (data not shown), an observation indicating the "robustness" of the method as specifically evaluated in the next section.

**Table 5:** Accuracy and reliability of QSS on viral DNA extracted from an infected plant

	**Replicate n°**																				
			
	**1**	**2**	**3**	**4**	**5**	**6**	**7**	**8**	**9**	**10**	**11**	**12**	**13**	**14**	**15**	**16**	**17**	**18**	**19**	**Average**	**SD**
**VIT 1**	42	40	42	41	41	41	40	41	41	44	42	44	44	43	43	42	43	42	43	42.0	1.3
**VIT 2**	5.4	5.6	5.5	5.8	5.9	6.6	6.0	5.8	6.1	4.9	5.6	5.2	5.2	5.1	5.3	6.6	5.8	5.3	5.4	5.6	0.5
**VIT 3**	39	36	40	38	37	39	39	40	39	41	39	41	42	42	42	38	40	42	42	39.9	1.8
**VIT 4**	11	12	11	11	12	11	11	11	11	9	11	9	8	9	9	11	9	9	9	10.3	1.2
**VIT 5**	1.4	3.3	1.1	1.9	2.5	1.3	2.2	1.3	1.2	1.1	1.4	0.6	ND	0.5	0.6	2.8	1.4	1.1	1.1	1.5	0.7
**VIT 6**	1.1	2.9	1.0	1.9	1.7	1.4	2.1	1.2	1.0	ND	0.7	ND	ND	ND	ND	ND	ND	ND	ND	1.5	0.6

Viral DNA was extracted from a plant infected with the CaMV Mix6VIT, and processed independently 19 times for PCR amplification and QSS analysis. Average final estimates (proportionally corrected to give a total of 100% in each case) and standard deviation (SD) among repeats are shown on the right.

Peaks yielded by low-frequency markers VIT 5 and VIT6, when identified, emerged just above the base line of the sequence traces. As indicated in the text, the corresponding frequency estimates reported in this table fall below the threshold of QSS accuracy, as confirmed by the high SD associated with VIT5 and VIT6, and by numerous repeats where estimates could not be obtained (ND), because the corresponding peaks were not clearly distinguishable on the electropherogram.

### Robustness of QSS on samples of sub-optimal quality

In the analysis of viral samples from the CaMV Mix6VIT-infected plant (Table 5), repetitions 1–11 and 12–19 were performed using 2 ng and 5 ng of viral DNA matrix, respectively, at the initial PCR amplification step. Presumably due to differences in the efficiency of the PCR, or for other unknown reasons, these two subsets yielded final trace sequences of different quality (not shown). Higher baselines in the former subset seemingly resulted in biased sums of marker frequency, which totalled between 116 and 137% before final correction (Table 6), whereas such biases were smaller in the latter subset, with total frequencies ranging from 110 to 117%. However, the final corrected estimates from both data subsets showed only very small differences in the mean frequency of markers, as well as in the standard deviation observed between repeats within subsets (Table 7). We thus conclude that, even on samples yielding trace sequences of sub-optimal quality, QSS still provides accurate and reproducible information on the genetic composition of a DNA population.

**Table 6:** Sum of the frequency estimates of all the markers before final correction

**Replicate n°**																			
**1**	**2**	**3**	**4**	**5**	**6**	**7**	**8**	**9**	**10**	**11**	**12**	**13**	**14**	**15**	**16**	**17**	**18**	**19**	
125	137	120	128	129	123	125	122	122	116	121	111	110	111	110	117	110	112	111	**Total amount (%)**

The sum of the frequency estimates of all markers before final correction is given here as an indicator of the quality of the sequence traces. Two subsets of differing quality can be distinguished (see text): replicates 1–11 (sums range from 116–137%), replicates 12–19 (sums range from 111–117%).

**Table 7:** Robustness of QSS on samples of sub-optimal quality

	**Average**		**SD**	
	**1–11 set**	**12–19 set**	**1–11 set**	**12–19 set**
**VIT 1**	41.2	43.0	1.1	0.7
**VIT 2**	5.8	5.5	0.4	0.5
**VIT 3**	38.8	41.3	1.3	1.3
**VIT 4**	11.1	9.2	0.7	0.6
**VIT 5**	1.7	1.0	0.7	0.8
**VIT 6**	1.5	ND	0.6	ND

Mean frequency estimates and SD for all markers within the two subsets shown in Table 6.

## Discussion

This report describes the development of a novel technique – Quantitative Single-letter Sequencing (QSS) – allowing simultaneous quantification of the frequencies of numerous individual allelic genetic markers in a mixed DNA population without the need for multiplexing adjustments. Although several techniques for the simultaneous detection and quantification of multiple variants at a single locus have been described previously [25-27], the QSS method has several advantages making it an interesting alternative. Implementation of QSS requires no specialized equipment, and it involves only a single PCR step and one sequencing reaction, allowing high throughput analysis. Two sequencing plates (96 wells each) were sufficient to establish all the standard curves in this study, and we were able to subsequently process hundreds of individual biological samples within a week. Due to these practical advantages, our method competes very well with easily accessible techniques based on real-time PCR [28], primer extension [26], quantitative sequencing [28], or RFLP analysis [29]. Indeed, the simultaneous monitoring of up to 6 CaMV variants afforded by QSS represents a significant progress, as other techniques require complex multiplexing setups that have thus far precluded efficient monitoring of more than 2 or 3 allelic variants in a single reaction tube. A concomitant study, recently published by another research group, used complex (four-letter) sequence electropherogram and statistical modeling to extract qualitative and quantitative information from bacterial DNA samples containing several allelic variants [30]. Remarkably, this study and QSS together confirm that methods based on quantitative sequencing can be applied to a wide variety of microorganisms. The two methods however have essential differences. QSS uses simple single-letter sequence traces, allowing the direct quantification of variants, exclusively from discriminating peaks, with no statistical modeling. Moreover, the use of fluorescent sequencing primer in QSS theoretically allows multiplexing in future development of the method. For instance, when two distinct polymorphic regions are present in the DNA sample, the use of two sequencing primers labeled with different fluorochromes can be envisaged, each targeting a specific locus. Such multiplexing, with two distinct unlabeled sequencing primers is not even theoretically possible with the use of dye-terminators (as in [30]), as the deciphering of overlapping sequences from the two distinct loci would be impossible.

The remarkable accuracy of the estimates obtained (MD and R^2^) and the very small standard deviation (SD) between repeats, make QSS equally, if not more, reliable than other techniques for which we could extract similar parameters from the literature (Table 8). We also tested the method on DNA samples of different quality (purified plasmid mixes versus viral DNA extracted from plants) and used variable amounts of initial template for PCR, and found that even in sub-optimal conditions yielding sequence traces with relatively high background or baseline, estimates of the genetic composition of the population were only slightly affected.

**Table 8:** Compared performance of QSS and other available methods

**Methods and associated references**	**MSD^a ^(%)**	**MD^b ^(%)**	**R^2^**
QSS	0.281 to 0.950	1.255	0.984 to 0.999
BAMPER [38]	3.8*	ND	0.9999
Micro-Array [39, 40]	3.5 to 4.1	2.4	0.971 to 0.9921
Micro-Satellite [41]	ND	ND	0.97
Mass Spectrometry [29]	1.55	ND	ND
PE+DHPLC [42]	1.4	1.2	0.977
Pyrosequencing [29, 43, 44]	0.07* to 1.9	ND	0.979 to 0.996
Quantitative Sequencing [28]	4.2	1.44	ND
RFLP [29]	2.8	ND	ND
RFMP [25]	ND	ND	0.992
Single base extension [26, 29]	0.27 to 1.75	1.5 to 2.15	ND
SYBR Green [28, 45]	1.65 to 6.47	1 to 1.12	0.997
TaqMan Probe [28, 29, 46]	0.75 to 3.18	1.47	0.9984

^a ^Reproducibility is evaluated by the median of standard deviations (MSD) among repeats on different variants, and is expressed as a frequency in %. For QSS, the overall MSD is 0.401%; the values shown here, 0.281 and 0.950% are calculated from Table 4 (plasmid mix) and Table 5 (viral DNA population extracted from plant), respectively.

^b ^Accuracy is estimated i) by the median deviation (MD), being the median of all differences between observed and expected allele frequencies, and ii) by the correlation coefficient between observed and expected frequencies (R^2^).

ND: Not-Determined

(*) these values refer to standard deviation (SD), because the data available in the literature do not allow calculation of the MSD.

During development of the QSS method, we observed an intriguing phenomenon (already mentioned above and presented in Table 2). When summing up the estimated relative frequency of all variants within a DNA sample, a value different from 100% was most often observed. We attribute this phenomenon, at least partly, to the fact that in most cases the baseline of the sequence traces is not exactly zero (not shown). Normalization of the height of the discriminating peaks to that of a peak common to all sequences (the normalizing peak) was intended to correct for this kind of fluctuation, but obviously did not completely do so when the baseline deviated significantly from zero. Moreover, attempts at normalizing discriminating peaks to any other common peak, positioned before or after the marker region, or even with an averaged height of several of these common peaks, did not significantly ameliorate this phenomenon (not shown). One explanation could be that the baseline is the same for both the normalizing peak and the discriminating peaks. Since the former is contributed to by all sequences within the population and the latter solely by a fraction thereof, the proportion of the total height of different peaks attributable to the baseline will be different. We believe that this leads to a slight overestimation of the discriminating peak, yielding sums above 100% when the baseline is above zero, with a possible distortion inversely correlated to the relative frequency of the genetic variant within the population. Although it may be possible to correct this undesirable artifact by further development of the QSS method, we show here that a simple proportional adjustment of all values to give a total of 100% is sufficient to provide an excellent approximation of true marker frequencies.

## Conclusion

QSS is very well suited to monitoring allele frequency changes in populations of pathogens such as viruses, and probably fungi or bacteria, in either single host extracts or pooled extracts from numerous hosts, provided that the polymorphism at the locus under surveillance is previously identified. It could also be applied to estimation of homo- or heterozygosity in di- or polyploid organisms, as well as to pools of DNA genomes extracted from numerous individuals to determine the relative frequency of variants within populations.

The genetic markers introduced into the CaMV genome in this study were not designed specifically for the development of QSS, and this has two theoretical implications. On the one hand, it suggests that designing markers specifically for QSS (i.e. several discriminating peaks per variant with appropriate spacing between each) would likely avoid partially overlapping peaks (such as those visible in Figure 1C) and thus allow monitoring of even more co-existing variants in a population. On the other hand, it shows that QSS can be applied to any non-specifically designed sequence variation, such as natural variants, again with the requirement that polymorphism at the corresponding locus is previously known. The method proved very efficient even with marker VIT3, where only one discriminating peak was available. Thus, the polymorphic region can either be very diverse, or bear only minimal sequence changes generating only a single discriminating peak.

## Methods

### Engineering markers in full-length CaMV clones

CaMV is the type member of the genus *Caulimovirus *and has a circular dsDNA genome of around 8000 bp, depending on the isolate. We used plasmid pCa37 [31], containing the full-length genome of the Cabb-S isolate of CaMV, to amplify viral gene II with primers P2Spe5' (5'-GGACTAGTATGAGCATTACGGGACAACCG-3') and P2Spe3'Killer (5'-AGCTCCTAGGTTAGCCAATAATATTCTTTA-3'). The resulting PCR product was digested with SpeI and AvrII, and cloned into plasmid pΔII-S at the unique *Spe*I restriction site separating ORFs I and III [32]. Reintroducing the PCR-amplified gene II into pΔII-S generated the full-length clone pCa-VIT0 retaining a unique SpeI restriction site between ORFs I and II.

Six different short dsDNA sequences of 40 bp each (generating the six distinct genetic markers shown in Figure 1A), with SpeI cohesive ends, were then inserted into the SpeI site in pCa-VIT0 (the sequences of the oligonucleotides used for generating these dsDNA markers are available upon request). The six clones obtained were named pCa-VIT1 to pCa-VIT6. All plasmids were purified using a Midiprep Kit (Qiagen), verified by sequencing, and stored in distilled water at -20°C until use.

### Quantifying pCa-VIT plasmids for PCR template preparation

Purified pCa-VIT1-6 plasmids stocks were thawed and carefully quantified by measuring the UV absorption at 260 nm of two dilutions for each plasmid (1 and 1/10), and duplicating each analysis in two different spectrophotometers (Varian Cary 50; Shimadzu UV-160A). A 1 mL solution, designated as the reference stock, was immediately prepared for each plasmid at a concentration of 50 ng·μL^-1^, and the concentration was verified again using the same two spectrophotometers before storage at -20°C. All single or mixed plasmid solutions subsequently used as PCR templates in the present study were prepared from these reference stocks.

### Infectivity and stability of CaMV-VIT clones

Turnip plants (*Brassica rapa *var. "Just Right") were maintained in an insect-proof greenhouse under controlled conditions (25/19°C day/night with a photoperiod of 16/8 hours day/night).

Seedlings at the three-leaf stage were mechanically inoculated with *Sal*I-digested plasmid (pCa37 or one of the pCa-VIT1-6 plasmids; 2 μg in 1× TE buffer per plant), as previously described [33]. Symptoms characteristic of systemic CaMV infection appeared between 14 and 21 days post inoculation (dpi) in all cases, indicating that the infectivity of the marker-containing CaMV-VIT1-6 clones was comparable to that of the wild type clone.

At 21 dpi, each CaMV-VIT1-6 virus clone was transferred to new healthy plantlets by mechanical sap inoculation. After two successive plant-to-plant passages (21 days each), viral DNA was extracted from single plants as described below, and the integrity of all six genetic markers was verified by sequencing, confirming their stability.

### Inoculation of plants with a mixed CaMV-VIT population

Leaves systemically infected with CaMV viral clones were collected at 21dpi, ground in buffer 1 (200 mM Tris, pH 7, 3v/w), and stirred overnight with 1.5 M urea and 2% triton-X-100 (final concentration). These crude extracts were then clarified by centrifugation at 3,000 × g for 15 min., and the supernatants were further ultra-centrifuged through a 15% sucrose cushion at 200,000 × g for 2 hours. The virus particles in the pellet were suspended in 600 μl of buffer 2 (100 mM Tris, pH7; 2.5 mM MgCl_2_), submitted to final clarification at 15,000 × g for 20 min. and stored at -20°C until use.

Equal volumes (200 μl) of virus particle preparations from plants infected with each of the 6 CaMV-VIT clones were pooled to produce a mixed population designated Mix6VIT. Note that we were aware that the different CaMV-VIT clones are not necessarily present in equal amounts in this mix at this point. Young healthy plantlets were then mechanically inoculated by carefully rubbing 20 μl of Mix6VIT on the total surface of three young leaves previously powdered with the abrasive carborundum. Symptoms indicative of CaMV infection appeared within 8 to 11 days, and all plants were considered systemically infected at 13 dpi.

For unknown reasons, CaMV symptoms always appear much earlier on plants inoculated with viral particles than on those inoculated with plasmid DNA, as in the previous section.

### Purification of viral DNA and PCR amplification

CaMV genomic DNA was purified from infected plants at 13 dpi according to the protocol described previously [34], plus an additional cleaning step using the Wizard DNA Clean-up kit (Promega).

PCR amplification of viral sequences was performed in a total volume of 100 μl, using VENT^® ^DNA Polymerase (New England Biolabs) according to the supplier's recommendations. Samples (2–5 ng) of plasmid mix or purified viral DNA were amplified by 35 PCR cycles, using the CaMV-specific primers F748 (5'-CTTGGAGCGGTCAAAATATTG-3') and Ris7 (5'-GTTGGGTACCTAAGGCTTCTAATATCTC-3'), generating a 1301 bp fragment spanning 601 and 660 bp upstream and downstream, respectively, of the introduced genetic marker. The primers were designed relatively far away from the polymorphic region to alleviate putative problems related to allele specificity during PCR amplification. The amplified DNA fragment was finally purified with a double phenol/chloroform extraction and ethanol precipitation, suspended in 45 μl water and processed for single-letter sequencing as described below.

### Single letter sequencing

The single-letter sequencing reactions were carried out by COGENICS Genome Express according to a proprietary protocol initially developed for DACS^© ^technology [35]. Briefly, the quality and quantity of each PCR product were assessed by agarose gel electrophoresis. The PCR products were diluted as required and sequencing reactions were performed with 13 fmol of template DNA. The dye-primer single-letter sequencing reaction was performed using the oligonucleotide 6FAM-1236bis (5'-ccttcaaataggtaacagtgc-3' linked to a 6FAM fluorochrome at its 5' end) and ddATP (0.1 mM final concentration) as the sole terminator to halt strand elongation [36], thus labeling only fragments terminated by adenine (A). [Note – In this study, we produced trace sequences only for the targeted base adenine (A), although theoretically any other nucleobase could be similarly targeted.] After sequencing, the reactions were purified by ethanol precipitation and suspended in formamide containing a labeled DNA ladder (GenScan 500 Liz; Applied Biosystems, Foster City, CA, USA). The single-letter sequencing signatures were analyzed on a capillary electrophoresis ABI 3730 DNA analyzer (Applied Biosystems) according to the manufacturer's instructions for DNA fragment analysis.

The 6FAM-1236bis sequencing primer was positioned 112 bases upstream of the marker region – a compromise between too long a distance engendering a decrease in fluorescence sequencing signals, and too short a distance increasing the risk of putative allele specificity problems. In our case, the electropherogram was exploitable without significant decrease in signal intensity from 75 to 200 bp downstream of the fluorescent sequencing primer.

### Extracting data from single-letter sequencing electropherograms

From the scanned images of the single-letter sequencing signatures, electropherograms were produced and analyzed using STRand – *Nucleic Acid Analysis Software *(freely available at [37]). The position of peaks as well as their height (directly provided by STRand in arbitrary units for the latter) were recorded for marker identification and quantification, respectively, as described in detail in Results.

## Authors' contributions

BM performed research except single-letter sequencing reactions which were carried out by HD. SB had the idea to exploit single-letter sequencing for distinguishing and quantifying several virus variants in a mixed population. BM, HD and SB extracted data from single-letter sequencing electropherograms. HD helped to draft the manuscript. BM and SB wrote the manuscript. All authors have read and approved the final version of this manuscript.
